# Medical and Physical Intelligence

**Published:** 1800-06

**Authors:** 


### MEDICAL and PHYSICAL INTELLIGENCE.

Prof. Hufeland diſtinguiſhes two ſpecies of vomition of the milk obſerved in infants. In the firſt, the milk is vomited up unaltered, a long time after it has been in the ſtomach; this is a ſymptom of weakneſs of the ſtomach, and want of digeſtion. The ſecond ſpecies is very different from the former; the child throws up the milk in a more or leſs coagulated ſtate, half an hour or a quarter of an hour after having taken it. This vomition is ſalutary, and gives proof of a ſtrong and powerful gaſtric liquor; by means of it, the ſuperabundant milk is brought up, and the reſt better aſſimilated. Againſt the former complaint, as well as againſt ſome other common affections of that infantile age, Mr. Hufeland recommends the following powder: Rec. Radic. Valerian dr. j. ireos florent. dr. jſs. liquirit. dr. ij. ſemin. anis. dr. jſs. crov. optimi gr. vijj. ſal. amar. dr. j. m. f. pulvis. two ſmall tea ſpoons full to be taken three times a day. When the convulſions are ſtronger, an addition of flor. zinc. or muſk, is uſeful; or ſome rhubarb, in caſe an evacuation ſhould be thought neceſſary. In a feveriſh ſtate, ſome tartarus vitriolatus, or nitre, may be added.—*Hufeland's Journal, Vol. ix. No. i. p.* 179.

Dr. Hargens, of Kiel, recommends in the Macula Corneæ, the external uſe of muriated barytes or terra ponderoſa ſalita, diſſolved in aqua Lauroveraſi, or diſtilled water of Prunus Lauroveraſus, L. The proportion is, terræ ponderos. ſalit. ſcrup. dimid. ſolve in aquâ Lauroveraſi unc. iij. Some drops of this ſolution to be dropped into the eyes as often as poſſible; every hour, &c. The patient feels ſome pain after it, which ceaſes in a few minutes. The diſtilled water of Lauroveraſus, by itſelf, is ſometimes very uſeful in chronic opthalmics with ſlight obſcurations of the cornea. *Ibid.*

The ſame gentleman has found the external application of hydrargyrus acetatus, or mercury prepared with acetous acid or vinegar, very uſeful in chronic exanthemata, particularly in ſuch as are of the flat, dry, herpetic, or ſerpiginous kind; but the general treatment muſt not be neglected. The ſhape in which he uſes it, is an ointment of two ſcruples and a drachm of hydrargyrus acetatus, tus, well mixed with one ounce of fresh butter, or spermaceti, or sweet oil; or a solution of 10 to 12 grains of mercury in 5 ounces of rose-water with a little mucilage of quince-seed. Of this, more or less to be several times a day applied on the eruption, till it dries away and disappears, and to continue it for some time after.

---

Two instances of Idiosyncrasy are mentioned by the same author; the one of a lady who was obliged to go to stool whenever she happened to sneeze repeatedly; a pinch of snuff had the same effect with her as a cathartic, The second, a gentleman, who got always a diarrhœa at any great, unexpected, or sudden noise from a shot, drum, &c. As he was often subject to costiveness, such an accident became sometimes very desirable to him. *Ibid.*

---

*Salivation from opium.*—An old woman fell into a considerable salivation after every dose of opium she took, in whatever form it was given. She had medical assistance for a great many ulcerations which covered her body; and whenever occasion required opium, or even any other narcotic medicine to be given, that effect was always produced, though in a less degree by the other narcotics, extract. hydrarg. nux vomica, &c. It is probable, that, as she had used a great quantity of mercury before, from quacks, who supposed her to be venereal, a part of it might have remained latent in her body, which was freed by opium and the other antispasmodic medicines, and then had its usual action. This is certainly very remarkable, as she had not taken mercury for twenty-two months before. An argument favourable to this conjecture is, that the salivation was stopped by liver of sulphur, the calx antimonis sulphurata, alkalis, by which all mercury is decomposed. *Ibid.*

---

Dr. Knebel recommends the liver of sulphur as a very useful remedy in some cases of struma combined with spongia usta; besides, a combination of spongia usta with gunpowder in equal parts, has had good effect. It is mixed with ten or twelve parts of dough and baked bread. Of this bread, three or four slices are given every morning and evening. *Materialien zur Argneywissenschaft.*

---

Professor Reiche, at Erlangen, has discovered a new arcanum, which he maintains to be an infallible remedy for all sorts of fevers, when there is not a principal fault existing in the organization. It is founded, at the same time, upon a new theory of fever. He offered to the King of Prussia, to make this remedy known for a certain sum of money. As Mr. Reiche is known as a man of learning and respectability, the King has accepted his offer, and sent for him to Berlin, to make experiments with this new remedy, and to see whether it will prove so as Professor Reiche asserts. A committee has been named, consisting of the most celebrated physicians at that place, viz. Dr. Selle, Dr. Fritye, Dr. Formey, and Richter, under whose inspection he is to try his practice, Of the patients patients who were trufted to him, feveral are faid to have died, others recovered. But as no particular accounts have yet appeared of the tranfaction of this bufinefs, authorized by thofe men, we think it proper to defer a relation to another time. Prof. Reiche is willing to communicate his arcanum to any phyfician for one guinea, for which he receives a paper confifting of half a fheet in quarto, printed in cyphers with a written key to it: but he defires every one to give him a bond, not to reveal the fecret to any perfon.

---

Citizen Grandchamp relates two curious inftances of preternatural offifications; the firft was a bony fubftance as big as a fift, fituated between the bladder and uterus, and inclofed in a proper fac, formed by the peritonaeum. The woman in whofe body it was found, had during her life an habitual ifchury, from which fhe only was relieved by an horizontal fituation on the back. This complaint might have been eafily miftaken for the ftone. The fecond obfervation is an uncommon offification of the gall bladder, found in the body of a woman of fixty-feven years of age. The place at the bladder was occupied by a hard mafs as big as the head of a fix or feven months fœtus; the ductus cyfticus was wanting, and the ductus hepaticus joined the liver immediately with the duodenum; the mafs itfelf cohered to the liver by a fmall place of the fize of a fmall coin. On opening it, a gluey fubftance was feen, fofter in the middle, and harder the more it approached to the periphery, till it gradually became offified. *Hufeland's Newfte Annalen; i. e. Newest Annals of French Medicine and Surgery, Vol. i. No. i.*

---

Citizen Buniva made fome experiments with injections into the blood-veffels of living and dead bodies. It is a known fact, that in fome difeafes, blood enters into veffels of the animal body, which do not receive it in a natural ftate. This phenomenon has been afcribed to a diffolution and putrefaction of the blood; an opinion confidered to be erroneous by modern pathology. However, we ftill wanted experiments to prove its being fo. The experiments inftituted to that end, are the the following: A mixture of calf's blood and water was injected into the veffel of a human and animal cadaver with fome force; it penetrated into the fmalleft veffels which, during the life of the animal, contain no blood. The fame injection was repeated in living animals, but it was impoffible to effect the fame phenomenon; the animal was killed at once, without the leaft lofs of blood, by cutting through the fpine; and the matter of injection immediately entered the fmall veffels. It is to be regretted, that thefe interefting experiments have not been more circumftantially defcribed; many queftions might be propofed, which now remain unfatisfied. *Recueil Periodique de la Societé de Santé, à Paris, An viii. Brum. p.* 110.

---

Citizen Vauquelin has found a new earth in the beryl from Siberia, whofe properties are the following: 1. It produces with acids faccharine, gently aftringent falts. 2. It is foluble in vitriolic acid, acid, without forming alum. 3. It is ſoluble in ammonia, which ſeparates it from the acids 4. It has an equal affinity to magneſia as to aluminous earth. It has received the name of glucine, on account of its forming ſweet ſalts with acids. *Journal de la Societé de Pharmacien de Paris.*

---

We have received a NOTICE from the Cow-Pock Inſtitution, by which the Public are informed that genuine Cow-pock matter may always be had there, under the ſeal of the Inſtitution.

---

Dr. BRADLEY will commence his Summer Courſe of Lectures on the Practice of Phyſic, at the Lecture Room, No. 102, Leadenhall Street, on Monday, June 2, at ſix o'clock in the afternoon.

---

Dr. BATTY will begin his Summer Courſe of Lectures on the Theory and Practice of Midwifery, and on the Diſeaſes of Women and Children, on Monday, June 9, at his houſe, No. 6, Great Marlborough-ſtreet, at eleven o'clock in the morning. Practical Midwifery as uſual.——N. B. This Courſe will be the only one given during the Summer.

